# A Biosorption Isotherm Model for the Removal of Reactive Azo Dyes by Inactivated Mycelia of *Cunninghamella elegans* UCP542

**DOI:** 10.3390/molecules17010452

**Published:** 2012-01-04

**Authors:** Sandra T. Ambrósio, José C. Vilar Júnior, Carlos A. Alves da Silva, Kaoru Okada, Aline E. Nascimento, Ricardo L. Longo, Galba M. Campos-Takaki

**Affiliations:** 1 Post-graduate Studies in Biological Sciences, Federal University of Pernambuco, 50.670-420 Recife, PE, Brazil; Email: sandra_ambrosio@hotmail.com; 2 Department of Fundamental Chemistry, Federal University of Pernambuco, 50.670-901 Recife, PE, Brazil; Email: longo@ufpe.br; 3 Nucleus of Research in Environmental Sciences, Catholic University of Pernambuco, 50.050-900 Recife, PE, Brazil; Email: carlos_vilar@hotmail.com (J.C.V.J.); calves@unicap.br (C.A.A.S.); elesbao@unicap.br (A.E.N.)

**Keywords:** *Cunninghamella elegans*, adsorption, textile dyes, isotherms, ultrastructure, biomass

## Abstract

The biosorption of three reactive azo dyes (red, black and orange II) found in textile effluents by inactive mycelium of *Cunninghamella elegans* has been investigated. It was found that after 120 hours of contact the adsorption led to 70%, 85%, 93% and 88% removal of reactive orange II, reactive black, reactive red and a mixture of them, respectively. The mycelium surface was found to be selective towards the azo dyes in the following order: reactive red > reactive black > orange II. Dye removal from a mixture solution resulted in 48.4 mg/g retention by mycelium and indicated a competition amongst the dyes for the cellular surface. A Freundlich adsorption isotherm model exhibited a better fit, thus suggesting the presence of heterogeneous binding sites. Electrondense deposits observed on the mycelium ultrastructure suggest that the dyes are mainly retained under the cellular surface of the inactive biomass of *C. elegans*.

## 1. Introduction

Reactive azo dyes are versatile colorants used by industries worldwide. The textile industry is an important and major sector in the Brazilian economy. However, it has a high potential to produce pollutants. Effluents, consisting of a complex mixture of colored and toxic compounds are generated from its processes. During processing, a significant amount of water, energy and chemicals is consumed, and around 10–20% of dyes are lost into the effluent during dyeing processes [1].

In addition, such effluent can reduce the penetration of sunlight into natural bodies of water, thus leading to a decrease of both photosynthetic activity and of the concentration of dissolved oxygen. The dye content of textile wastewater can be large due to losses of up to 50% dye during secondary textile processes [1,2,3].

The presence of dyes in watercourses is both aesthetically unacceptable and possibly toxic inasmuch that a genotoxic effect on organisms tends to be associated with contamination. As a result, the appropriate biotreatment of textile effluents is very important nowadays [4,5,6]. However, treatment can be difficult due to the synthetic origin and the complex aromatic molecular structures of the most common dyes, which are by design chemically stable and therefore not easily biodegradable [7]. 

The treatments of dyes in wastewaters can be classified as: (*i*) destructive, which leads to the partial or total degradation of the dye molecules; and (*ii*) non-destructive, which eliminates the color by transferring the molecules of the dye to some appropriate (solid) supports [8,9,10,11]. In this latter class of the process, the adsorption of such supports has been quite efficient, and thus widely investigated. However, due to the chemical nature of the surface of the activated carbon, the adsorption of cationic dyes is not as efficient, this therefore being an important limitation of this technique [12]. On the other hand, there is great potential for exploiting fungal mycelium for treatment of textile effluents in which dye in the wastewater is bioadsorbed by inactive mycelium [13,14]. There are many advantages to using inactivated mycelium such as selectivity, high removal rates, high removal ratios, low costs, and regenerative potential [15]. A comparison of living and inactive mycelium shows that inactive mycelium can be easily stored and used for long periods, while live mycelium requires nutrients and is subject to several physiological restrictions, strict operational and conditions of maintenance [16]. As to the removal of dye from textile effluent by live mycelium, it is important to distinguish between dye degradation by fungal metabolization and the adsorption of dye. The efficiency of the biosorption process, under equilibrium conditions, of reactive azo dyes by *Rhizopus arrhizus* has been shown to be more efficient than active carbon [17]. This efficiency is attributed to the interactions between the characteristics of the reactive group of textile dyes and those of the active groups on the surface of the fungal cells [18]. Pre-treatment methods lead to binding sites and increase the interaction between the dye and hyphae which induces the decolorization process. The process is related to the number of active sites for adsorption, and can be explained by the increase in electrostatic interactions between the ionic groups in the cell walls and the polar or ionic dye molecules. In acidic solution, the basic groups of the cell wall can be protonated and thus become binding sites for anionic groups or molecules such as reactive dyes [19]. In any case fungal biomass is considered a good or excellent adsorbent [20,21,22]. This paper puts forward the use of isotherms in a model of adsorption by mycelia, and evaluates the decolorization of three reactive azo dyes and their mixtures by the inactive mycelium of *Cunninghamella elegans*.

## 2. Results and Discussion

### 2.1. Kinetics of Adsorption of Azo Dyes

**Figure 1:**
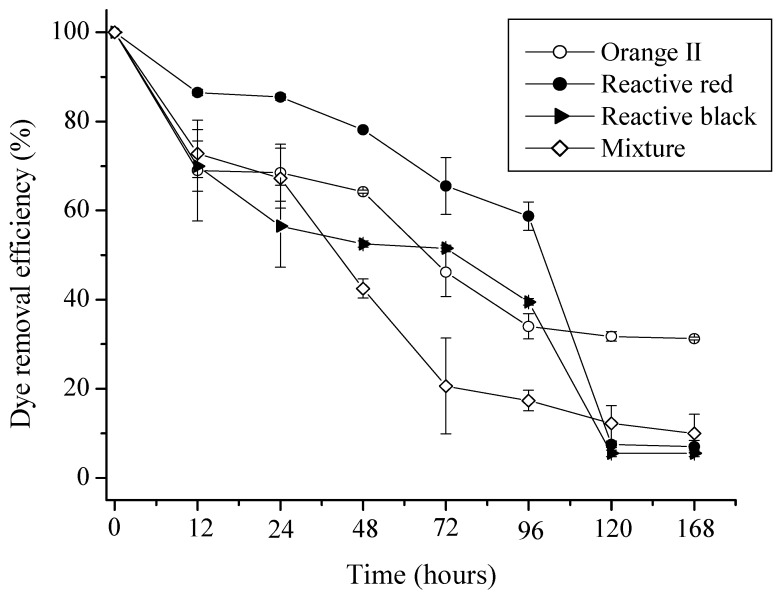
Decolorization kinetics for the azo reactive dyes and their mixture by inactivated mycelium *C. elegans*.

Regarding the adsorption kinetics, it was found that contact time has a strong influence on the biosorption of orange II, black-5 and red-198 and their mixtures. 30% decolorization was observed in the first 12 hours of contact, except for red-198 dye. However, after 120 hours of contact the colors decreased to 85%, 93% and 88% for the black-5, red-198 dyes and their mixture, respectively. We observed decolorization of the solutions containing orange II dye was smaller, namely, around 70%, and after 72 hours this became constant. The decolorization profiles of black and red dyes were similar, possibly due to the similar number of anionic groups in their molecular structures. Similar profiles were observed for the mixtures of dyes, probably due to the predominant uptake of black and red reactive (Figure 1).

As the cell walls of the inactivated biomass of *C. elegans* used in our study mainly contain chitin and chitosan, high adsorption rates are an expected result. Chitin is a polymer of *N*-acetyl glucosamine, and chitosan is produced from the deacylated form of chitin. The dye-binding kinetics of chitin are very slow, unfortunately, requiring five days to reach equilibrium [23].

Many industrially useful fungi contain chitin and chitosan in their cell walls. Hence, the fungal biomass byproducts of industrial fermentation processes can serve as an alternative source of chitin-based dye adsorbents. The results obtained are consistent suggesting the behavior of inactivated biomass of *C. elegans chitin* and chitosan binding to azo dyes.

A similar type of mechanism was postulated to explain the adsorption of 60% of textile dye Gris lanaset G by mycelium inactivated using sodium azide of *Trametes versicolor*, and may also explain the high adsorption of reactive dyes onto activated carbon. On the other hand, the influence of the length of contact time on biosorption can be vary in accordance with the characteristics of the dye and microorganism [24], and this observation is corroborated by the results obtained in our study using *C. elegans*.

### 2.2. The Langmuir and Freundlich Isotherms

**Figure 2:**
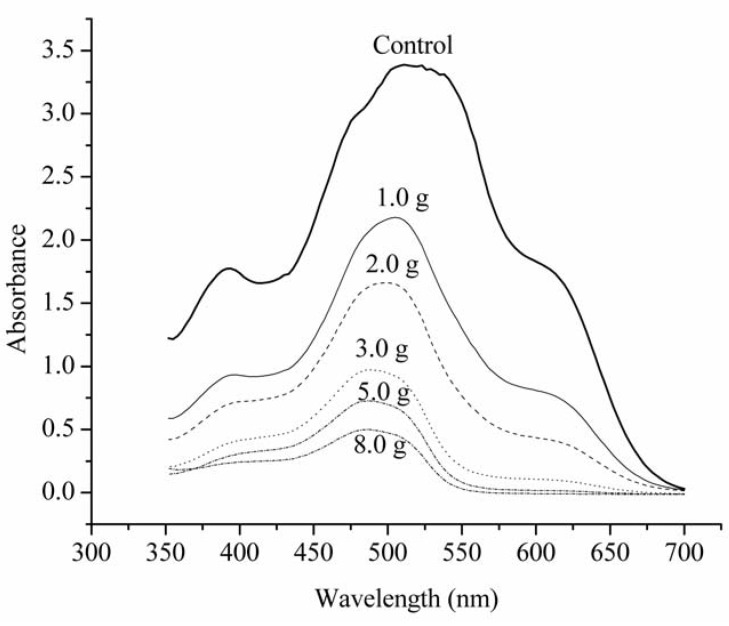
UV-Vis spectra of the dye mixture after 120 hours of contact with inactivated mycelium *C. elegans* as a function of the adsorbent mass from top to bottom: control (without the mycelium), 1.0, 2.0, 3.0, 5.0 and 8.0 g.

The Langmuir and Freundlich adsorption models were used for the mathematical description of adsorption and were obtained from UV-visible absorption spectra (Figure 2) measurements.

The Langmuir model is valid for monolayer sorption onto a surface with a finite number of identical sites [16,25] and is expressed by:

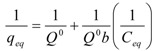
(1)
for which the notation is given in Table 1.

The empirical Freundlich model is based on sorption on a heterogeneous surface [9] and is expressed as:

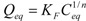
(2)


**Table 1:** Notation used in the adsorption isotherms.

*b*	Langmuir affinity adsorption constant (mg/L)
*C_eq_*	Concentration at equilibrium of non-adsorbed dye (mg/L)
*C_0_*	Initial dye concentration (mg/L)
*K_F_*	Proportionality Freundlich adsorption constant
*n*	Exponent Freundlich adsorption constant
*q_eq_*	Amount of adsorbed dye per unit weight of mycelium at equilibrium (mg/g)
*Q^0^*	Limiting Langmuir adsorption constant (mg/g)
*R^2^*	Linear correlation coefficient
*X*	Mycelium concentration (g/L)

Table 2 presents the characteristics of the constants of the isotherms of adsorption (*Q*^0^ and *b*; *K_F_* and *n*) for each dye and for the mixture of azo dyes. The Freundlich isotherm yielded a better linear fit (linear regression coefficients, *R*^2^, larger than 0.99) than the Langmuir model (*R*^2^ > 0.95), thus suggesting that the adsorption surface presents heterogeneity. The mycelial surface of *C. elegans* was observed to be more selective towards the red dye, followed by reactive black and orange II. At equilibrium, the mycelium adsorbed 23.5, 4.18 and 6.94 mg/g of red, black and orange dyes, respectively, and 48.4 mg/g of the mixture of dyes. However, the adsorption affinities of the individual dyes from the mixture were smaller than from a single component solution (see Table 2). 

**Table 2:** The Langmuir and Freundlich adsorption isotherms for the azo reactive dyes and their mixture on the inactivated mycelium *C. elegans* at 28 °C.

	**Langmuir Model**			**Freundlich Model**		
Dyes	*Q*^0^ (mg/g)	*b* (mg/L)	*R*^2^	*K_F_*	*n*	*R*^2^
Orange	6.94	1.52	0.983	3.71	2.43	0.993
Red	23.5	2.41	0.980	6.86	2.16	0.996
Black	4.18	1.96	0.980	2.71	1.37	0.995
Mixture	48.4	0.20	0.980	10.5	2.23	0.995

This is probably due to competitive adsorption [13], to the interaction between the dyes and to the changes of the adsorbent surface charge caused by the adsorption. This competitive adsorption is responsible for the large amounts of the orange II dye remaining in the solution, since its binding affinity is smaller than for the other dyes [26]. It should be noted that, remazol yellow was the first dye adsorbed on the surface of active F-400 carbon, reducing the amount of available adsorption sites and thus the uptake of black and red dyes [12,24]. As a result, it is suggested that the active carbon and inactive mycelium of *C. elegans* are complementary with respect to their selectivity in adsorbing azo reactive dyes. As mentioned before, the better fit of the adsorption data by the Freundlich isotherm suggests some heterogeneity of the binding sites, possibly due to modifications of the functional groups resulting from the inactivation process [9], and to the intrinsic physiology of the microorganism [24].

From Table 2, it can be seen that the adsorption by *C. elegans* has 1/*n* values varying from 0.41 to 0.73. For comparison, it was found that the value of 1/n varies from 0.5–1.4 for biosorption on microbial cells depending upon their genus and the adsorbed organic compound. In addition, it is usually accepted that adsorbent with 1/*n* values between 0.1–0.5 are considered efficient and if 1/*n* > 2.0, they are considered to be inefficient [27]. Accordingly, the inactive mycelium of *C. elegans* can be considered a good adsorbent. Some species of *Aeromonas* are also good biosorbents of reactive textile dyes such as red and black remazol [28], while *R. arrhizus* displays favorable adsorption with black remazol.

Decolorization due to the adsorption of dyes upon different amounts of inactive mycelium can be quantified by the UV-Vis spectra as illustrated in Figure 2. Examination of these spectra reveals that all absorption bands decreased proportionally to the amount of biosorbent and there are no spectral differences between the control and the solution after treatment. This indicates that the dye molecules are not being degraded and confirms the adsorption process on the cellular surface of the cell [29].

Although many properties of solids are known in great detail, the nature of their adsorption sites is still not well known, especially with regard to cellular surfaces. For filamentous fungi, in general, the polysaccharides presented in the cellular walls, when exposed by the inactivation process can increase adsorption significantly [30]. For instance, in *Aspergillus niger*, in addition to the functional groups of the cell surface and the chemical structure of the dyes being strongly dependent upon the pretreatments imposed on the biosorbent so too is the mechanism of the biosorption of the dyes, since the mechanism can increase the adsorption capacity by exposing latent binding sites [18]. It is also known that chitin and chitosan occur in the cell wall of fungi and have a high biosorption capacity. Considering the predominant presence of these polysaccharides in the cell walls of Zygomycetes, the taxonomic class within which *C. elegans* is included, it can hypothesized that these two chemical groups might be the major contributors to the biosorption of the reactive azo dyes studied [30,31,32].

### 2.3. Ultrastructural Analyses

**Figure 3:**
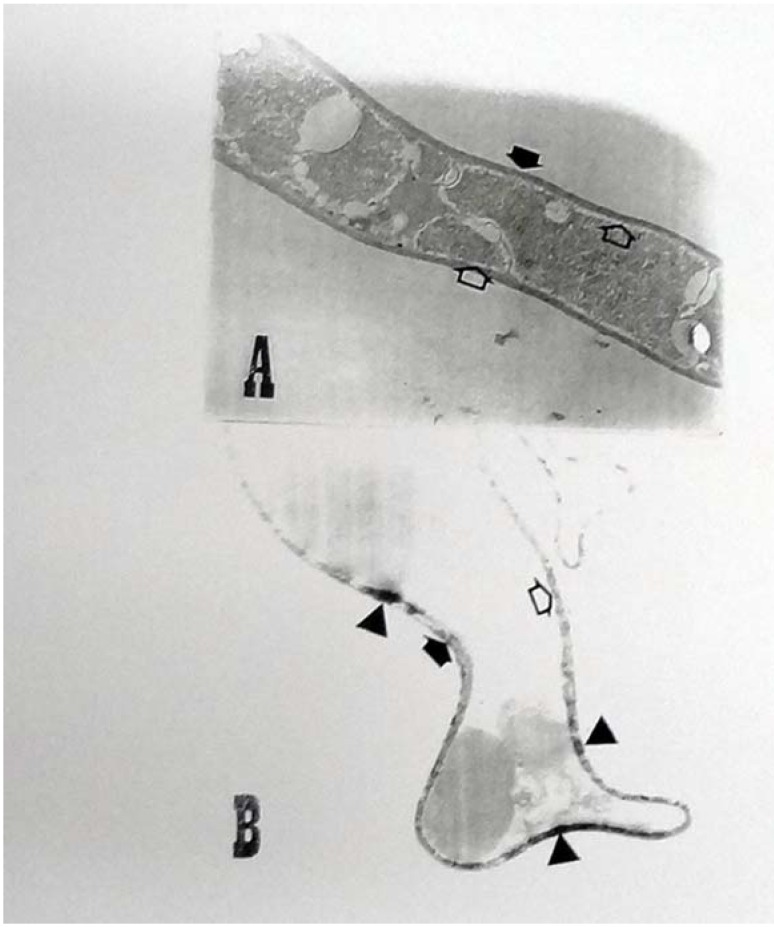
Electron micrographs of *C. elegans*. (**A**) Control mycelium 10.000× enlargement and (**B**) inactive mycelium after biosorption of the black reactive 14.000× enlargement. Solid arrows indicate cell walls, open arrows the citoplasmatic membrane, solid triangle the electrondense deposits.

The results obtained from ultrastructural analyses demonstrated alterations to the strain *C. elegans* after biosorption of dyes associated with the texture and homogeneities of cytoplasm, and the presence of electron-dense aggregates on the cell surface. The occurrence of these electron-dense aggregates suggests the accumulation of adsorbed dyes in comparison with control samples. Despite this, it can be suggested from the electron microscopy analyses that fine structure alterations on the surface of the cell walls after the biosorption of dyes had occurred (Figure 3A and Figure 3B).

The elucidation of some mechanistic aspects of the biosorption of metal ions through the cell wall of *Phanerochaete chrysosporium* was conducted by ultrastructural analyses [27,33]. Also, regarding the biosorption of metal ions, it was shown by undertaking ultrastructural analyses of the cell surface of *Aspergillus niger* that pretreatment in order to inactivate the mycelium increases the adsorption capacity [18,34]. An excellent ability to degrade reactive textile dyes is demonstrated by Brazilian strains of *T. villosa* and *P. sanguineus* [35]. *Aspergillus fumigatus* XC6 isolated from mildewing rice straw was capable of decolorizing dye effluent over a pH range 3.0–8.0 with the dyes as the sole carbon and nitrogen sources.

Therefore, *A. fumigatus* XC6 is an efficient strain for decolorizing reactive textile dye effluents, and it might be a practical alternative in treating dye in wastewater. However, the adsorption process is not limited to the external side of the cell wall. These results have also a profound impact on the distinction between degradation and adsorption for the decolorization process by inactivated biomass, such as by the potential sorbent from *Cunninghamella elegans*.

## 3. Experimental

### 3.1. Microorganism

*Cunninghamella elegans* UCP 542 was isolated from mangrove sediments, and was kindly supplied by the Culture Collection of the Nucleus of Research in Environmental Sciences, Catholic University of Pernambuco (Recife, PE, Brazil), included in the Network of microorganisms in the North and Northeast of Brazil-RENEBRA, and registered in the World Federation Culture for Collection-WFCC. The culture was maintained on Sabouraud dextrose agar at 4 °C. The liquid medium used for large-scale biomass production was maintained on Sabouraud dextrose agar for 5 days at 28 °C. 

### 3.2. Dyes

Table 3 presents some features of the azo dyes studied. The azo dye orange II (C.I. 15510) was obtained from Sigma and the C.I. reactive black-5 and reactive red-198 (DyeStar) were provided by Suape Têxtil Co., Cabo, Pernambuco, Brazil.

**Table 3:** Dye information: molecular mass–MM (g/mol), wavelength maximum absorption–λ_max_ (nm).

Dyes	MM	l_max_	Classification	Chemical structure
Orange II	350	485	Anionic monoazo	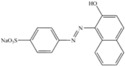
Black-5	992	597	Anionic diazo	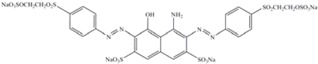
Red-198	1470	517	Anionic monoazo	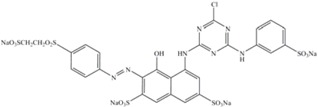

### 3.3. Biomass Preparation

The fungal cultures were grown in 250 mL Erlenmeyer flasks using sporangiole suspensions (3.8 × 10^8^ sporangioles/mL) in 95 mL of Sabouraud liquid medium, the glucose of which was replaced by sucrose, considering the previous studies of the literature [12,35], and incubated in an orbital shaker at 150 Hz. After a period of 72 hours, the biomass was collected by vacuum filtration.

### 3.4. Mycelium Inactivation

The mycelium was inactivated by using 1% formaldehyde solution [36]. After inactivation, the mycelium was placed in a shaker (100 Hz, at 28 °C) for 2 hours, and then filtered and submitted to liofilization overnight. Subsequently it was re-suspended and homogenized in a shaker (150 Hz, at 28 °C) for 2 hours. The mycelia were harvested by filtration through a silkscreen nylon filter (0.8 µm).

### 3.5. Adsorption Kinetics

The inactivated mycelium was added to 100 mL of culture media in 500 mL Erlenmeyer flasks containing one of the dyes (0.025 mM) or a mixture of them (0.034 mM). The pH of the medium was adjusted to 5.6 by using phosphate buffer (0.1 M) before sterilization at 121 °C, and the cultures were incubated in an orbital shaker (150 Hz, at 28 °C). The kinetics of decolorization was studied by collecting 2 mL of aliquots at 12, 24, 48, 72, 96, 120 and 168 hours. Control experiments were prepared without the mycelium and all the experiments were carried out in triplicate.

### 3.6. Adsorption Isotherms

Different amounts of mycelium, 0.1, 0.2, 0.3, 0.4, 0.5, 0.6, 0.7 and 0.8 g, were added to 500 mL Erlenmeyer flasks containing 100 mL of culture medium and a solution of dye mixture (0.034 mM). The experiments were conducted in an orbital shaker (150 Hz) at 28 °C and samples were collected at 168 hours after the addition. The uptake of the dyes in solution was determined by using the procedure described in the literature [37]. The notation employed in the isotherm expressions is presented in Table 2. All experiments were carried out in triplicate.

### 3.7. Color Reduction Measurements

Absorbance measurements were performed with a Spectronic Gênesis, model 2 UV-Visible spectrophotometer. The wavelengths for the measurements were set at the values shown in Table 3 and the dye concentrations were calculated from an absorbance and molar concentration calibration curve. The calibration curves presented linear ranges of 0.005–0.025 mM and 0.005–0.034 mM and linear regression coefficients larger than 0.99.

### 3.8. Ultrastructural Analysis

Samples were washed twice in PBS (phosphate-buffered saline) at pH 7.2 for 10 minutes and fixed in 2.5% glutaraldehyde in 0.1 M cacodylate buffer at pH 7.4 for 2 hours at room temperature. They were then post-fixed in 1.0% of osmium tetroxide for 1 hour at room temperature. Post-fixation was followed by washing with a 0.1 M cacodylate buffer at pH 7.4. Samples were dehydrated in acetone and embedded in Epon and cut in a REICHERT-JUNG ultramicrotome, collected on copper grids and contrasted with uranyl acetate and lead citrate. Electron micrographs were obtained using a JEOL 100-CX (80 Kv) transmission electron microscope [38].

## 4. Conclusions

This paper put forward a model-based evaluation of the process of biosorption of reactive dyes using the inactivated mycelia of *Cunninghamella elegans*. Fungal biomass is an industrial fermentation byproduct can serve as an alternative biosorbent. Ultrastructural investigations are scarce, but the model produced an important tool for evaluating the sites to which azo dyes attach themselves during the decolorization process. Biosorption is an environmentally friendly and efficient process, and a potential low cost method for removing chemical azo dyes from textile effluents.
